# Effects of carnosine supplementation on physical endurance: a placebo-controlled randomized clinical trial

**DOI:** 10.1080/15502783.2026.2679716

**Published:** 2026-06-17

**Authors:** Timothy E. O'Toole, Christopher J. Wingard, Sonja K. Bariess, Catherine E. Crandell, Alok R. Amraotkar, Hong Gao, Aruni Bhatnagar

**Affiliations:** a Department of Medicine, Christina Lee Brown Envirome Institute, University of Louisville, Louisville, KY, USA; b Department of Medicine, Division of Environmental Medicine, University of Louisville, Louisville, KY, USA; c Department of Physical Therapy, Bellarmine University, Louisville, KY, USA

**Keywords:** Carnosine, physical performance, endurance, clinical trial

## Abstract

**Background:**

Carnosine (β-alanine-L-histidine) is an endogenous dipeptide found in abundance in highly metabolic tissues such as skeletal muscle and the brain, where it is thought to play a role in intracellular buffering, and thereby, promote anaerobic glycolysis. Due to its perceived ability to support muscle energetics, carnosine, and its precursor, β-alanine, have found widespread use as performance-enhancing supplements. Nevertheless, the utility and efficacy of carnosine in enhancing physical performance have not been rigorously evaluated.

**Methods:**

We conducted a placebo-controlled clinical trial in which 299 participants were randomized to consume either placebo or carnosine-containing capsules (2 g per day). Several assessments of physical performance (hand grip strength, bilateral calf raise, 2-min step test, gait speed) were measured before initiating supplementation (Baseline: Visit-2) and after approximately 6 week (Visit-3) and 12 week (Visit-4) of supplementation. At each clinical visit, blood and urine were also collected. For Visit-3 and Visit-4, absolute changes and percent changes from baseline were calculated for each physical function measurement.

**Results:**

At Visit-3 we observed a statistically significant (*p* = 0.018) increase in the calf raise measure (number of bi-lateral flexions to exhaustion) for those in the carnosine supplementation group younger than 40 years of age. At Visit-4 we observed a statistically significant (*p* = 0.010) increase in the number of steps for males in the carnosine supplementation group greater than 40 years age and a trend toward significance (*p* = 0.06) for all males taking carnosine. No significant (*p* < 0.05) differences were observed in the carnosine versus placebo supplementation groups for any other physical performance measure.

**Conclusions:**

In select age groups, carnosine supplementation improves muscular and cardiorespiratory endurance but not grip strength. The benefits appeared to be limited to males. Carnosine supplementation is likely to have beneficial effects in those undertaking short-term repetitive movements.

## 1. Introduction

Carnosine (β-alanine-L-histidine) and its biochemically modified derivatives comprise a family of histidyl-containing dipeptides found in abundance in highly metabolic tissues (skeletal muscle, heart, brain) of all species [1]. Due to its unique chemical structure, carnosine participates in a range of biochemical reactions that support its role in maintaining tissue homeostasis. Among its several biochemical roles, carnosine is particularly efficient in sequestering hydrogen ions and buffering intracellular pH, although it can also chelate transition metals, quench reactive oxygen species, and bind to carbonyls, thereby neutralizing the effects of lipid peroxidation and glycation [1]. In part due to such diverse roles, both clinical and animal studies have found that increasing carnosine levels can protect from ischemic injury [2,3], neurodegenerative disease [4], toxic outcomes of air pollution exposure [5], and promote wound healing [6], glucose handling [7,8] and cognitive function [9–14].

Because the levels of carnosine are particularly high in skeletal muscle, and because intense physical activity induces intracellular pH changes and oxidative stress, carnosine has been widely promoted as a supplement to enhance athletic performance. Nevertheless, studies on the effects of carnosine on physical performance have given somewhat mixed results. While some have shown that increasing skeletal muscle carnosine content enhances the capacity for high-intensity exercise [15–17], others have shown little benefit [18–20]. The reasons for these discrepant results are unclear, but they may be reflective of the type of physical exercise which was assessed, cohort differences, or differences in supplementation protocols. Furthermore, most of these earlier studies used cohorts of rather limited size and demographics, cohorts of highly trained athletes, or somewhat extreme dosing regimens (≥5 g per day) [15,21–23].

To more clearly define the impact of carnosine in physical performance, we analyzed seven indices as secondary outcomes measures of participants in the Nucleophilic Defense Against PM Toxicity (NEAT) clinical trial [24], a randomized, placebo-controlled study to examine the impact of carnosine supplementation on biomarkers of cardiovascular health, physical health, and cognition. It is one of the largest carnosine supplementation studies to date, using a cohort of diverse ethnicities, ages, and physical activity levels. It used a dosing regimen (2 g per day) with demonstrated historical efficacy and limited side-effects [17,25]. Carnosine uptake and distribution in this cohort was well-characterized [26]. In our analysis of physical performance we observed that carnosine improved muscular and cardiovascular endurance in select age groups. Males appeared to derive the most benefit. Thus, supplementation with carnosine or its precursor, β-alanine, may prove beneficial for mostly short-term repetitive movements.

## 2. Methods

### 2.1 Study cohort

A complete description of the NEAT trial including overall design, enrollment criteria, supplementation strategy, clinical measures, and data analysis approaches has been published [24]. Of the 299 participants who qualified and were randomized for the study, 283 returned for Baseline (Visit-2) physical performance and clinical measurements. At this time the participants were also supplied with carnosine or placebo (corn starch) capsules which were identical in appearance and instructed to consume these supplements (2 × 0.5 g, twice per day; total of 2 g per day). Two return clinical visits (Visit-3 and Visit-4) after approximately 6 and 12 weeks of supplementation were scheduled for biospecimen collection and repeat physical performance and clinical measures. Unused supplements were counted at return visits to assess compliance. Only data from those participants with ≥80% compliance was included in the final analysis. Also excluded from final analysis were carnosine non-responders who were defined as those in this supplementation group whose urinary carnosine levels increased less than 10% of their baseline values. This trial was carried out in accordance with The Code of Ethics of the World Medical Association, has Institutional approval (University of Louisville IRB: #20.0258; Bellarmine University IRB: #901), and is registered at ClinicalTrails.gov (NCT03314987). All participants gave informed consent to be included in the study.

### 2.2 Physical performance measures

The physical functions measured are listed in Table S1 and have been described in greater detail elsewhere [24]. The hand grip strength static test was assessed as described using a Jamar Technologies Plus + Digital Hand Dynamometer (JLW Instruments) [27]. For each participant, an initial maximum contraction was determined as the highest of three individual contractions. After a one-minute rest, the participants were instructed to squeeze the dynamometer in a static contraction at a level of 50% of their maximal contraction for as long as possible with verbal feedback from the examiner. The participants then performed a final post-fatigue maximal contraction within 10 s of completion of the 50% maximal static contraction. These measurements were made on both hands. For the calf raise test, the participants performed the maximum number of plantar flexions possible to a pre-determined height (based on a single maximal plantar flexion), as fast as possible until the point of fatigue. Measurements include the number of plantar flexions performed, total time in seconds, and repetition rate (repetitions per second). For the two-minute step test, the participants were asked to stand next to a wall and march in place with the intent of raising their knees to a marked target on the wall, representing a mid-way height between the lateral femoral condyle and anterior superior iliac crest of the participant. The number of times the right knee reached the marked target over a 2-min period was recorded. Finally, for gait speed, upon given cue, the participants walked as quickly as possible for a distance of 4 meters. Time in seconds was recorded, an average calculated over two repetitions, and a rate (m/s) calculated.

### 2.3 Statistical analysis

Demographic characteristics of the study group were presented as mean ± standard deviation for age, and frequency and percent for sex and race. At baseline, we examined the associations of age (≤40 years old; >40 years old) and sex with the seven physical performance measures. Differences between the placebo and carnosine supplementation arms were evaluated in the same way. Change in physical performance measures from Baseline to Visits 3 and 4 were determined both as an absolute change and as a percent change from baseline in both the placebo and carnosine arms. Eight subgroup comparisons were made (carnosine vs. placebo in: males and females; all participants ≤ 40 yr; all participants > 40 yr; males ≤ 40 yr; males > 40 yr; females ≤ 40 yr; females > 40 yr). A Student's *t*-test was used to identify significant differences between the groups. A similar analysis was conducted after stratification by age and sex. To further examine the effects of carnosine supplementation at Visits 3 and 4, mixed-effects models were constructed with physical performance measures as dependent variables and supplementation (carnosine or placebo) as the independent variables. In these mixed-effects models, we adjusted for age and sex. Estimated fixed effect coefficients (β) for the treatment (carnosine vs. placebo with placebo as the reference) against the seven physical function measures were calculated. Statistical significance was set at the *p*-value < 0.05. All analyses were performed using SAS, version 9.4 (SAS Institute, Inc., Cary, North Carolina). The forest plots were produced in Graph Pad Prism, version 9.1 (Graph Pad Software, La Jolla, California).

## 3. Results

### 3.1 Cohort characteristics

Of the 527 individuals screened for inclusion in the study, 299 qualified for randomization. From this group we ultimately obtained usable physical performance measurements for 283 participants at Baseline, 198 participants at Visit-3 (40.3 ± 4.6 days of supplementation), and 192 participants at Visit-4 (77.4 ± 5.7 days of supplementation) (Figure S1). There were similar participant demographics in the placebo and carnosine arms at all Visits (Table 1). As reported previously, the dose and duration of carnosine supplementation was effective in increasing endogenous levels, as reflected by an approximate 2-fold increase in erythrocyte levels and 7-fold increase in urinary levels of carnosine [26].

**Table 1.** Demographics of study participants.

Characteristic	Visit-2 (baseline)		Visit-3		Visit-4	
	Placebo (*n* = 136)	Carnosine (*n* = 147)	Placebo (*n* = 101)	Carnosine (*n* = 97)	Placebo (*n* = 101)	Carnosine (*n* = 91)
Age (yr) mean ± SD	44.7 ± 12.2	45.5 ± 12.3	46.1 ± 12.2	46.7 ± 12.7	45.6 ± 12	48.2 ± 12
Sex – *n* (%)						
Male	59 (43.4%)	64 (43.5%)	43 (42.6%)	39 (40.2%)	43 (42.6%)	45 (49.5%)
Female	77 (56.6%)	83 (56.5%)	58 (57.4%)	58 (59.8%)	58 (57.4%)	46 (50.5%)
Race – *n* (%)						
White	108 (79.4%)	122 (83.0%)	82 (81.2%)	84 (86.6%)	80 (79.2%)	81 (89.0%)
Other	28 (20.6%)	25 (17.0%)	19 (18.8%)	13 (13.4%)	21 (20.8%)	10 (11.0%)

Age is presented as mean ± standard deviation (SD), while the other characteristics are presented as frequency (%). Sex and race were self-reported.

### 3.2 Carnosine supplementation and physical performance measures

At Baseline, there were no differences in any of the seven measurements of physical performance among individuals who were more than, or less than, 40 years of age (Table 2). However, as expected, values for calf raise, step test, and all measurements of hand grip strength were higher in men than in women. Measurements of gait speed were similar between the two sexes. Moreover, none of the measures of physical strength in participants in the placebo arm were different from those in the carnosine arm, indicating that the baseline values in physical performance were similar between the two supplementation groups.

**Table 2.** Baseline values.

Variable	Age-dependence			Sex-dependence			Supplementation group		
	≤40 yr (*n* = 120)	>40 yr (*n* = 163)	*p*	males (*n* = 123)	females (*n* = 160)	*p*	Placebo (*n* = 136)	Carnosine (*n* = 147)	*p*
Hand grip Initial max-R	85.1 ± 24.7	85.0 ± 24.7	0.98	106 ± 20.7	69.4 ± 13.2	<0.001	85.2 ± 23.3	85.0 ± 25.9	0.95
Hand grip Initial max-L	82.8 ± 23.5	83.9 ± 26.8	0.72	105 ± 20.8	66.9 ± 13.2	<0.001	83.13 ± 24.7	83.7 ± 26.1	0.84
Hand grip Final max-R	75.0 ± 23.0	77.3 ± 24.3	0.43	94.6 ± 20.4	61.8 ± 14.5	<0.001	75.8 ± 22.9	76.8 ± 24.6	0.73
Hand grip Final max-L	70.2 ± 21.9	74.6 ± 23.4	0.12	90.3 ± 19.9	59.0 ± 13.7	<0.001	72.3 ± 21.9	73.0 ± 23.7	0.78
Calf raise	1.05 ± 0.37	1.05 ± 0.38	0.93	1.12 ± 0.4	1.00 ± 0.34	<0.01	1.08 ± 0.36	1.02 ± 0.38	0.25
Step test	100 ± 17.3	96.3 ± 21.3	0.08	101 ± 18.3	95.8 ± 20.6	0.03	98.8 ± 20.6	97.3 ± 19.0	0.55
Gait speed	1.30 ± 0.21	1.29 ± 0.24	0.85	1.32 ± 0.22	1.27 ± 0.23	0.08	1.29 ± 0.21	1.3 ± 0.24	0.93

Listed are the values for the physical function variables measured at Baseline (Visit-2). Stratification was by age (≤40 yr; >40 yr), sex (males; females), and supplementation group (placebo; carnosine). R = right; L = left.

**Figure 1.**
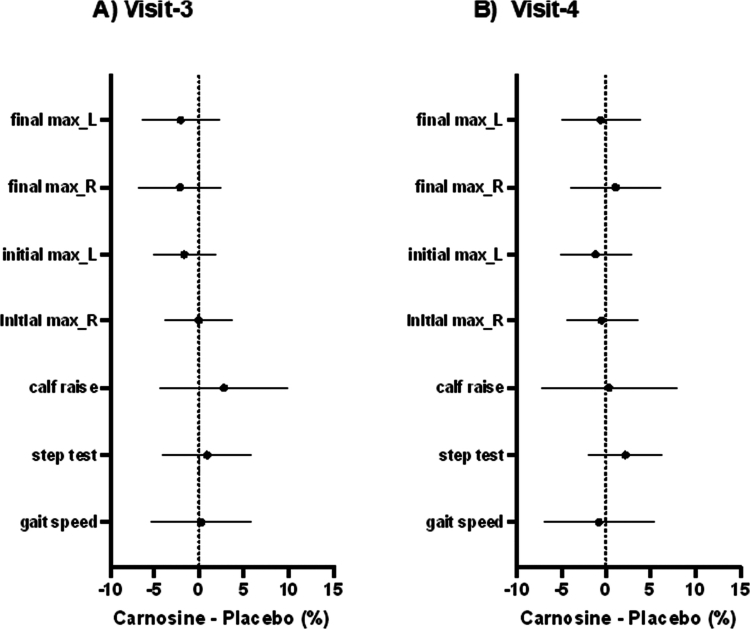
Changes in physical function measures. Illustrated are the percent change differences from Baseline, between the carnosine and placebo groups at Visit-3 (A) and Visit-4 (B).

**Figure 2.**
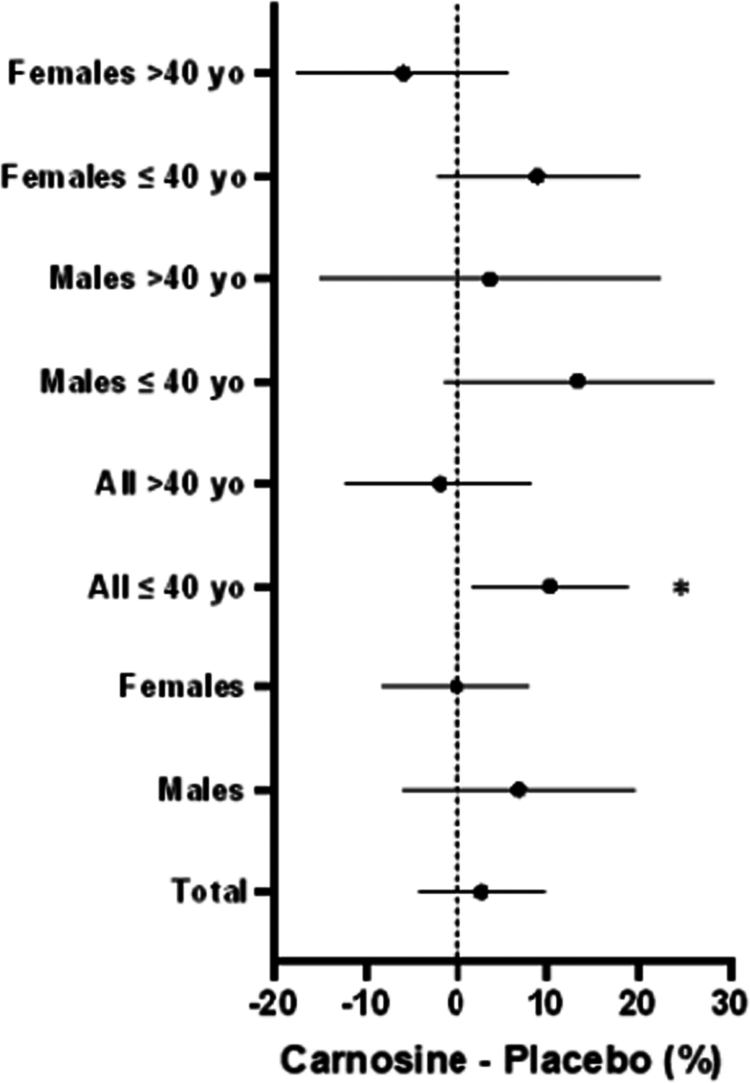
Changes in bilateral calf raise measures at Visit-3. Illustrated are the percent changes from Baseline for the listed groups. *p < 0.05.

At Visits 3 and 4, there were no differences in any of the measures between the treatment and the placebo arms, as the percent changes from Baseline for all participants in the two arms were not significantly different (Figure 1). For the hand grip measures, no differences were observed when this analysis was performed either after stratification according to right-left (Figure 1) or dominant-non-dominant hands (not shown). However, when we stratified the study group by age (≤40; >40) and sex, we observed that those in the carnosine arm ≤ 40 years of age had a statistically significant (*p* = 0.018) increase in the calf raise measure (Figure 2; Table S2). This corresponds to an average increase of 10.2% (95% confidence interval: 1.6%–18.9%) relative to the placebo group. The increase in this age group seemed to derive mostly from males (*p* = 0.078) rather than females (*p* = 0.339) (Table S2).

**Figure 3.**
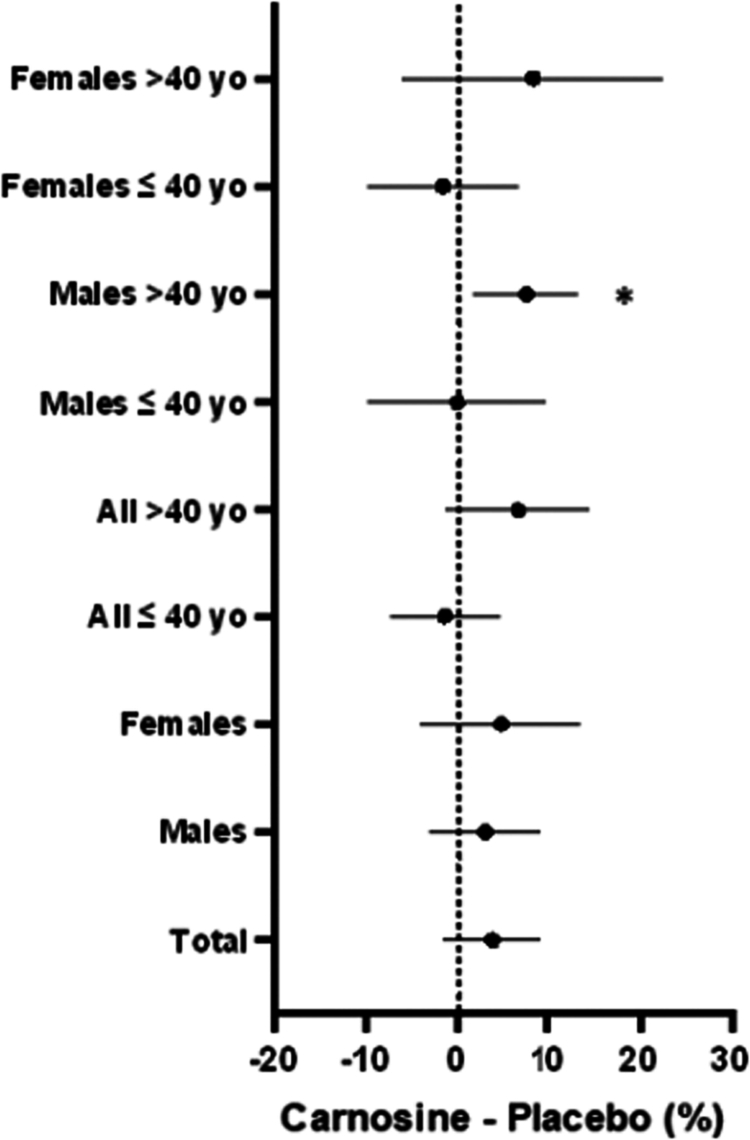
Changes in step test measures at Visit-4. Illustrated are the percent changes from Baseline for the listed. groups. *p < 0.05.

Percent changes from Baseline were also calculated after measurements at Visit-4. As for Visit-3, we observed no significant differences between the placebo and carnosine groups in any measure in the whole study group (Figure 1). However, after stratification, there was a trending increase (*p* = 0.06) in the step-test measures for all males (Table S3) and a significant increase (*p* = 0.01) in those males > 40 years of age (Figure 3; Table S3). This reflected on average, a 7.4% increase (95% confidence interval: 1.6%–13.2%) relative to the placebo group. When analyzed using fixed effects models for each of the performance measures after adjusting for age and sex, no differences were observed in the measured values (Table S4), suggesting that the effects were not age or sex-independent and were limited to specific age and sex groups.

## 4. Discussion

In a well-powered, randomized, placebo-controlled trial, we found that oral supplementation with carnosine improves endurance in specific tests of physical performance. For calf-raises as well as the step test, selective benefit seemed to occur in male participants of the trial. Although few studies have been conducted with carnosine itself, it has been previously reported that β-alanine ingestion also improves performance. However, most of these studies are limited by small sample sizes. Of the 46 studies listed in a collated meta-analysis of the effects of β-alanine, none exceeded a sample size of 30 [28], whereas in another, more recent analysis [29], the 25 studies listed all had sample sizes of less than 45 participants Most of these prior studies also used high doses of β-alanine (between 4 and 6.4 g/day) for a short duration (usually 4 weeks). In part because of such limitations, the results of these studies have led to variable, often contradictory results. Moreover, small sample sizes preclude subgroup analyses, so the demographics of potentially sensitive individuals could not be identified. Additionally, even in studies reporting positive effects, the effect sizes are indeterminate due to large variance. Thus, our randomized trial, with 283 participants and a prolonged treatment (6–12 week) with low-dose carnosine (2 g/day), represents the most well-powered attempt to examine the effects of carnosine on performance, and therefore, our observation that carnosine supplementation improves aspects of physical performance is based on the most rigorous evidence available to date.

Because β-alanine is a substrate for the *in vivo* synthesis of carnosine, supplementation studies have frequently administered this essential component. Results from these studies suggest a rather nuanced impact of increased carnosine levels on physical function. Consistent with the findings here that supplementation increased endurance, other studies have found that β-alanine increased time to exhaustion and anaerobic capacity in targeted groups such as trained cyclists [30–32], elite rowers [15], and strength trainers [33]. However no changes in muscle endurance were observed in young rugby players following a similar interventional regimen [20]. In other groups, β-alanine increased the endurance of knee extensor muscles [34], and generally attenuated fatigue in repeated muscle contractions [17]. However, these benefits of carnosine seem to pertain to short-term exertion exercises, as there was no correlation between its levels and improvement in long-term cycling episodes [19] or prolonged exercise [18]. The benefits of β-alanine ingestion in certain tests of short-term military operations were also observed, but only after supplementation with rather large doses [35,36]. In contrast to endurance, β-alanine has been reported to have no impact on skill acquisition or technical performance [37], although these conclusions are limited by a small sample size (*n* = 27), short duration of the trial (6 week), and the lack of efficacy evaluation (whether β-alanine supplementation led to an increase in carnosine levels). Similarly, we did not find an increase in strength as measured in the hand grip tests.

In general, the ability of carnosine to improve repetitive, high-intensity and short-term exercise rather than strength seems to be a common finding [38,39]. This effect of carnosine in improving time to exhaustion is likely due to its buffering capacity. Because it has an imidazole ring, carnosine can accept hydrogen ions, neutralizing pH and the effects of accumulated lactic acid which arise from short-term anaerobic exercise [32]. However, carnosine has diverse biochemical properties which may also lend to this protection, including it anti-oxidant and anti-glycating capacities as well as its ability to chelate certain metals [40,41].

While our analysis identified improvements in time to exhaustion in the calf raise and step test, these measures reflect different types of physiological endurance. The calf raise is a measure of muscular endurance [42] and in particular, for the plantar flexor muscles with strong interrater and test-retest reliability [43]. The generalized indicators for difference in strength would be reflected in ~32–38 repetitions for the general population [43–45], while men and younger individuals performed better [46]. The protective effect of carnosine in this scenario is likely due to it pH buffering capacity. We found the benefit of carnosine supplementation in the calf raise was most apparent in younger individuals This observation is similar to our recent finding that carnosine supplementation improved cognitive function in younger, rather than older individuals [9]. One potential explanation for this outcome could be that age is associated with an increase in the extent of oxidative stress and generation of lipid peroxides, and because carnosine can form adducts with these byproducts and eliminate them through urinary excretion, available carnosine stores are depleted due to this activity. Thus, we suggest that limited amounts of the dipeptide are available to exert ergogenic effects. In contrast, younger individuals, with fewer oxidative byproducts, have may have larger supplies of carnosine available for skeletal muscle pH buffering.

The 2-min step test can be used to assess aerobic capacity and has found general use in assessments of hypertensive, obese, or generally older adult populations. The test is known for its strong validity and interrater reliability [47] as a measure of cardiorespiratory endurance [48] and can be used to predict peak VO_2._ [49] These measures may be dependent upon cardiac levels of carnosine. Although we have found that increases in urinary and erythrocyte levels of carnosine plateau by 6 weeks of supplementation and increased little thereafter [26], the kinetics of cadiac accumulation are unknown and this may require more than 6 week. Furthermore, in the heart, significant levels of carnosine are present in an acetylated form (N-acetylcarnosine) [50], and the dynamics of carnosine acetylation are not known and may be a limiting factor in increasing myocardial levels of histidyl dipeptides. These uncertainties in cardiac accumulation may account for the observation that improvements in the step test were observed at Visit-4, but not Visit-3. As with carnosine, N-acetylcarnosine is a multifunctional peptide and can support cardiac function by several mechanisms [50]. However, levels of N-acetylcarnosine decrease with age [51], and it is older individuals with depleted stores, rather than the younger individuals who may be protected with more abundant endogenous supplies, who demonstrate greater benefit from carnosine supplementation in the step test. These results are similar to those previously demonstrating benefits to cardiac fitness in an older population taking β-alanine supplements [21].

We found that the effects of carnosine supplementation were sex-specific. In the step test we observed that older males, but not females had a benefit from carnosine supplementation. This sex-specific result appears to be in contrast to the reported beneficial effects in older females undertaking β-alanine supplementation [32]. However that study cohort involved competitive masters athletes, who may have lower endogenous levels of oxidative stress and lipid peroxides. Indeed, physical training and exercise are associated with decreased oxidative stress and inflammation [52]. Thus, carnosine stores in such individuals are more available for pH buffering and neutralizing lactic acid accumulation, and therefore the beneficial effects of carnosine may depend upon the level of training and fitness of an individual [28]. Interestingly, in the NEAT cohort, which was assembled independent of physical activity status, we did observe that older females, rather than males, had higher levels of urinary carnosine-propanal, which is indicative of oxidative stress [26]. Thus, it may not be unexpected that males, with apparently lower levels of oxidative stress, demonstrate a greater benefit from supplementation than females. Interestingly, β-alanine supplementation in a cohort of individuals presenting with chronic obstructive pulmonary disease, who also have significant levels of oxidative stress, did not improve time to exhaustion [53].

From the number of measures at baseline to those at Visit 4, we had 38% attrition in the carnosine arm of the study. While some of this loss can be attributed to problems arising during the measures themselves (failed quality control (QC)), the bulk of this loss can be attributed to participant withdrawal from the study for personal reasons (*n* = 13), individual non-compliance or individual non-responsiveness. Based upon results from prior studies, we expect that daily carnosine supplementation should increase its levels by at least 10%. Failure to attain these levels may be apparent in individuals who express unusually high endogenous levels of the carnosine- degrading enzyme, carnosinase. A classification of non-responsiveness may also result from those individuals who removed supplement capsules from their packaging (thus, classified as compliant) yet failed to ingest them. In the placebo arm of the study, we had somewhat lower attrition (26%) from baseline to Visit 4. While the number of individual withdrawals from this study group was similar (*n* = 11) to that in the carnosine arm, the smaller attrition is mostly due to the fact that non-compliance of placebo supplementation was not considered and non-responsiveness could not be calculated in this group. Thus, we contend there was randomness in the treatment arms with respect to the missing measures. We did not evaluate the data with an intent-to-treat protocol. Another limitation of the study is the lack of quantification of carnosine levels in skeletal muscle. While this has been previously done by mass spectrometry using biopsy samples [54] or by proton magnetic resonance spectroscopy in intact muscle [15,55], when used in large cohort studies, these procedures present financial and logistical concerns and are difficult to implement. Furthermore, there are technical concerns with these approaches [55,56]. However, we have previously demonstrated that urinary and erythrocyte carnosine measures may also be reflective of overall content and that approaches using these samples are valid and can be more practically applied [56].

In summary, in a well-powered, placebo-controlled randomized clinical trial, we found that supplementation with carnosine improved physical performance, an effect that was largely limited to males. While other studies have demonstrated similar benefits of β-alanine supplementation, they have generally used trained athletes, younger individuals, excessive dosing regimens, or had limited cohort sizes. Our results show this benefit can be attained in a population of a wide age-range and irrespective of physical fitness status.

## Supplementary Material

Supplementary Material Supplemental_information.docx
