# Supplementary material for: Effects of carnosine supplementation on physical endurance: a placebo-controlled randomized clinical trial
Source: J Int Soc Sports Nutr. 2026 Jun 17;23(1):2679716. doi: 10.1080/15502783.2026.2679716 (PMC13276806; doi:10.1080/15502783.2026.2679716)

**Effects of Carnosine Supplementation on Physical Endurance: a Placebo-Controlled Randomized Clinical Trial.**

**Supplemental Information:**

Table S1: Physical Performance Measures in the NEAT Cohort

Table S2. Calf raise: Visit-3

Table S3. Step test: Visit-4

Table S4 Mixed effect model

Figure S1. Participant enrollment and physical function measures

| **Table S1. Physical Performance Measures in the NEAT Cohort** | | |
| --- | --- | --- |
| **Measure** | **Description** | **Quantification (units)** |
| Hand grip strength | Initial max-right hand | (pounds) |
|  | Initial max-left hand | (pounds) |
|  | Final max-right hand | (pounds) |
|  | Final max-left hand | (pounds) |
| Bilateral calf raise | Plantar flexions to a pre-determined height | number of flexions and time to exhaustion (repetitions/sec) |
| Step test | 2-minute marching in place | number of times knees reach a certain height (number of repetitions) |
| Gait speed | Time to walk 4 meters | average of 2 repetitions (m/sec) |

Listed are those indices of physical function measured in the NEAT study.

| **Table S2. Calf raise: Visit-3** | | | | | |
| --- | --- | --- | --- | --- | --- |
|  | **Effect size** | | **Change from baseline** | | |
| **Group** | **coefficient** | **95% CL** | **placebo** | **carnosine** | **p** |
| **all** | 0.028 | -0.043 – 0.099 | 0.09 ± 0.28 | 0.13 ± 0.22 | 0.334 |
| **all ≤40yr** | 0.102 | 0.016 – 0.189 | 0.06 ± 0.19 | 0.17 ± 0.18 | 0.018* |
| **all>40yr** | -0.020 | -0.124 – 0.083 | 0.10 ± 0.32 | 0.11 ± 0.23 | 0.924 |
| **males** | 0.068 | -0.060 – 0.196 | 0.08 ± 0.31 | 0.17 ± 0.25 | 0.199 |
| **males≤40yr** | 0.133 | -0.015 – 0.281 | 0.03 ± 0.21 | 0.16 ± 0.17 | 0.078 |
| **males>40yr** | 0.037 | -0.151 – 0.225 | 0.11 ± 0.35 | 0.17 ± 0.30 | 0.509 |
| **females** | -0.001 | -0.084 – 0.081 | 0.10 ± 0.26 | 0.11 ± 0.18 | 0.973 |
| **females≤40yr** | 0.089 | -0.023 – 0.200 | 0.11 ± 0.24 | 0.17 ± 0.18 | 0.339 |
| **females>40yr** | -0.060 | -0.177 – 0.058 | 0.10 ± 0.29 | 0.07 ± 0.17 | 0.597 |

Listed are the effect sizes with 95% confidence limits (CL), and percent changes from baseline for

the calf raise measures at Visit-3 in the indicated groups. *:p<0.05.

| **Table S3. Step test: Visit-4** | | | | | |
| --- | --- | --- | --- | --- | --- |
|  | **Effect sizes** | | **Changes from baseline** | | |
| **Group** | **coefficient** | **95% CL** | **placebo** | **carnosine** | **p** |
| **all** | 0.036 | -0.018 – 0.091 | 0.07 ± 0.16 | 0.09 ± 0.14 | 0.57 |
| **all ≤40** | -0.015 | -0.075 – 0.045 | 0.07 ± 0.19 | 0.03 ± 0.11 | 0.31 |
| **all>40** | 0.065 | -0.015 – 0.145 | 0.08 ± 0.15 | 0.12 ± 0.14 | 0.15 |
| **males** | 0.029 | -0.032 – 0.090 | 0.03 ± 0.12 | 0.07 ± 0.12 | 0.06 |
| **males≤40** | -0.001 | -0.100 – 0.097 | 0.03 ± 0.16 | 0.03 ± 0.09 | 0.98 |
| **males>40** | 0.074 | 0.016 – 0.132 | 0.02 ± 0.09 | 0.10 ± 0.12 | 0.01* |
| **females** | 0.046 | -0.043 – 0.135 | 0.11 ± 0.19 | 0.10 ± 0.15 | 0.67 |
| **females≤40** | -0.017 | -0.099 – 0.064 | 0.07 ± 0.19 | 0.03 ± 0.11 | 0.31 |
| **females>40** | 0.081 | -0.062 – 0.224 | 0.08 ± 0.15 | 0.12 ± 0.14 | 0.15 |

Listed are the effect sizes with 95% confidence limits (CL), and percent changes from baseline

for the step test measures at Visit-4 in the indicated groups. *:p<0.05.

| **Table S4 Mixed effect model** | | | |
| --- | --- | --- | --- |
| **Measure** | **coefficient** | **95% CL** | **p value** |
| Gait speed | 0.022 | -0.013 - 0.056 | 0.220 |
| Step test | 2.019 | -1.170 - 5.208 | 0.214 |
| Calf raise | -0.058 | -0.117 - 0.002 | 0.057 |
| Initial max-R | -1.203 | -3.726 – 1.320 | 0.349 |
| Initial max-L | -0.023 | -2.513 -2.467 | 0.986 |
| Final max-R | -1.686 | -6.199 – 2.827 | 0.463 |
| Final max-L | -0.058 | -2.495 – 2.379 | 0.963 |

Listed are the fixed effect coefficients with 95% confidence limits (CL)

for the treatment (carnosine vs. placebo with placebo as the reference)

for the physical function measures estimated using mixed effect models,

adjusted for age and sex.


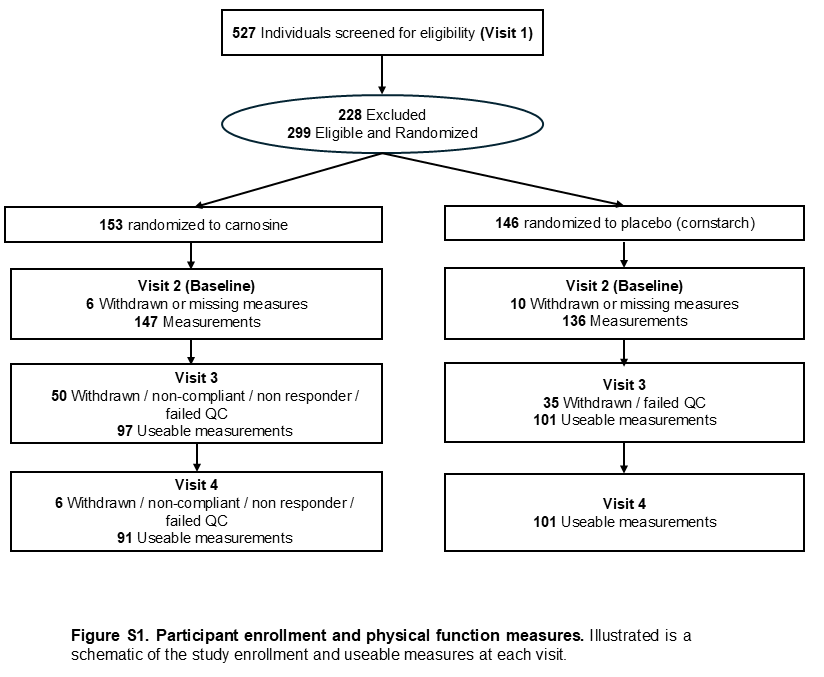

Supplement: Supplementary Material — Supplemental_information.docx [file RSSN_A_2679716_SM2920.docx]
